# Propofol differentially modulates the consolidation of schema-related and -unrelated memory

**DOI:** 10.1016/j.isci.2025.113415

**Published:** 2025-08-21

**Authors:** Lukas V. Risse, Deetje Iggena, Lili Landerer, Mario Menk, Heidi Olze, Daniel J. Salchow, Carsten Finke, Yee Lee Shing, Christoph J. Ploner

**Affiliations:** 1 Department of Neurology, Charité - Universitätsmedizin Berlin, 13353 Berlin, Germany; 2 Department of Anesthesiology, Charité - Universitätsmedizin Berlin, 13353 Berlin, Germany; 3 Department of Ophthalmology, Charité - Universitätsmedizin Berlin, 13353 Berlin, Germany; 4 Department of Otorhinolaryngology, Charité – Universitätsmedizin Berlin, 13353 Berlin, Germany; 5 Department of Psychology, Goethe University Frankfurt, 60629 Frankfurt/Main, Germany; 6 Center for Individual Development and Adaptive Education of Children at Risk (IDeA), 60323 Frankfurt/Main, Germany

**Keywords:** Pharmacology, Neuroscience

## Abstract

Semantic relatedness of newly learned information to previous knowledge (i.e., a schema) leads to facilitated encoding and rapid integration into neocortical memory networks. The precise dynamics of this process in humans is still poorly understood. Here, we used the GABA-A-ergic anesthetic propofol to transiently suppress neural activity shortly after the encoding of schema-related and -unrelated verbal information in human patients. We found a significant difference in memory of schema-related and -unrelated words in patients that was absent in controls. This effect was driven by a benefit for schema-related words, thus suggesting that propofol administration facilitated the consolidation of previously encoded schema-related information. Our results suggest that schema-relatedness of newly learned information significantly influences the involvement of brain networks shortly after encoding. They further support the hypothesis of a competitive interaction between networks supporting schema-related and -unrelated memoranda during early memory consolidation.

## Highlights

• Suppression of neural activity with propofol affects early memory consolidation
• GABA-A-ergic suppression can facilitate the consolidation of schema-related memory
• Distinct dynamics for the consolidation of schema-related and -unrelated memory

## Graphical abstract

**Figure:**
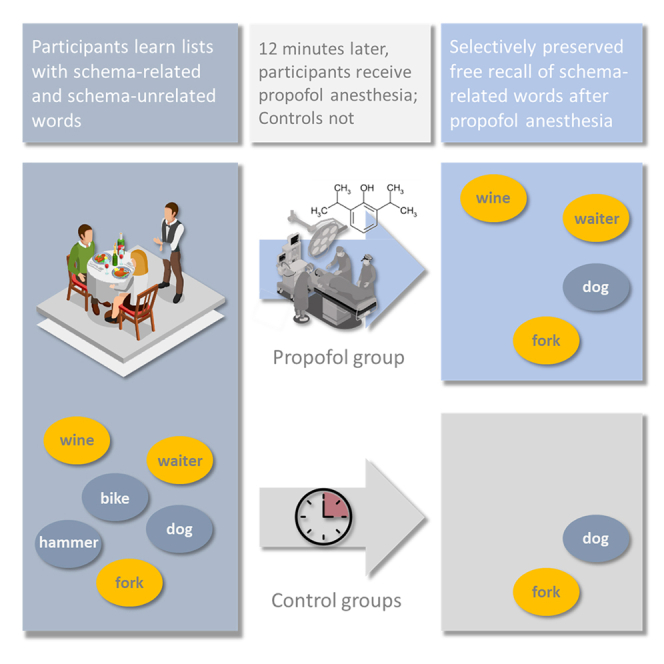

## Introduction

Theories on systems memory consolidation converge on the idea that the passage of time alters how the brain represents conscious memories.^1^^,^^2^^,^^3^^,^^4^ There is, however, an ongoing debate on the respective roles of different brain regions during systems consolidation. The heterogeneity of findings from studies suggests that memory consolidation is not a unitary process that stereotypically follows encoding but rather results from the interaction of distributed representations whose contributions are modulated by content-related and contextual factors.^2^^,^^3^^,^^4^ One important factor may be the semantic relatedness of new information to previous knowledge or mental schemas.^5^^,^^6^ There is evidence from animal and human studies that these factors facilitate the integration of new information into memory networks.^7^^,^^8^^,^^9^ In humans, EEG studies have shown that schema effects are already detectable at the encoding of new information into memory.^10^ fMRI studies have further shown that the availability of a schema significantly modulates connectivity between brain regions such as the hippocampus and ventromedial prefrontal cortex at encoding and during the post-encoding period.^11^^,^^12^^,^^13^^,^^14^ Neural activity during this latter period may represent an important step in early memory consolidation as it predicts later memory performance across a variety of tasks and stimulus materials.^15^^,^^16^ So far, it is, however, unclear whether neural activity in the post-encoding period differentially contributes to the consolidation of schema-related and -unrelated information.^8^

The investigation of the role of brain networks for early memory consolidation ideally requires techniques that modulate neural activity in neocortex and in deep brain regions such as the hippocampus without affecting prior encoding and later retrieval. However, these latter regions are not directly accessible for current non-invasive brain stimulation techniques. The invasive deep brain stimulation of the human entorhinal cortex during memory tasks is limited to patients undergoing evaluation for epilepsy surgery, and non-invasive electrical stimulation of the hippocampus is currently still in development.^17^^,^^18^ An alternate approach to interfere transiently with neural activity is the administration of the short-acting anesthetic propofol (2,6-diisopropylphenol). This drug acts as an agonist on the gamma-aminobutyric-acid (GABA)-A receptor and as a partial antagonist on N-methyl-D-aspartate (NMDA) receptors.^19^^,^^20^ GABA-A receptors play a suppressive role in learning and memory and are widely distributed in the brain, including the neocortex and hippocampus.^21^^,^^22^^,^^23^ Their activation with propofol tampers excitatory/inhibitory balance of cortical neurons and disrupts the brain’s capacity for information processing.^24^ PET and fMRI studies also showed that propofol significantly reduces whole brain glucose metabolism and suppresses hippocampal activation during memory tasks.^25^^,^^26^ Accordingly, in two recent studies, human participants learned verbal or visuospatial material and received general anesthesia with propofol some minutes thereafter. After recovery from anesthesia, participants showed a memory pattern that the suggested impairment of early steps of memory consolidation.^27^^,^^28^

In the current project, we combined the propofol approach established in previous work^27^^,^^28^^,^^29^ with a verbal schema memory task.^10^^,^^30^^,^^31^ In these tasks, the presentation of a category stimulus (i.e., a schema) is typically followed by blocks of stimuli that are either semantically related to the category or not. Subjects are requested to judge by button-press about the schema-relatedness of stimuli. In most studies, schema-relatedness accelerates encoding and enhances later recall of stimuli.^10^^,^^30^^,^^31^ Here, we used a new task variant that was adapted to the specific context and time-constraints of a pre-surgical setting. Furthermore, we aimed at ecological validity by randomizing stimuli that are semantically related to each other and a single pre-determined schema with an equal number of semantically unrelated stimuli from multiple categories. We reasoned that in naturally occurring contexts, most items that need to be remembered are semantically related to a distinct schema. For example, we rarely experience an equal number of items related to the schemas “hospital” and “restaurant” when we are in a restaurant. During the learning phase, no explicit judgements about schema-relatedness were required, since the positive confirmation of schema-relatedness may itself facilitate subsequent memory.^30^^,^^31^ Instead of an explicit category (schema) cue, a soundscape was provided during the learning phase that was semantically related to half of the stimuli. The soundscape was introduced to further facilitate the use of a mental schema in an easy and non-stressful way for participants.^32^^,^^33^

In our experiment, neurologically normal patients undergoing minor surgery learned the list of randomized words immediately prior to propofol anesthesia while being exposed to the soundscape via headphones. About 12 min after learning, participants received anesthesia with intravenous propofol for about 60 min. Three hours after learning, participants were tested for recall and recognition of schema-related and -unrelated words. In order to control for pre-surgical stress, performance was compared to participants receiving local anesthesia and to participants receiving no anesthesia. We reasoned that any differences in the early consolidation of schema-related and -unrelated words should lead to differences in memory performance following anesthesia.

## Results

### Learning

**Figure 1:**
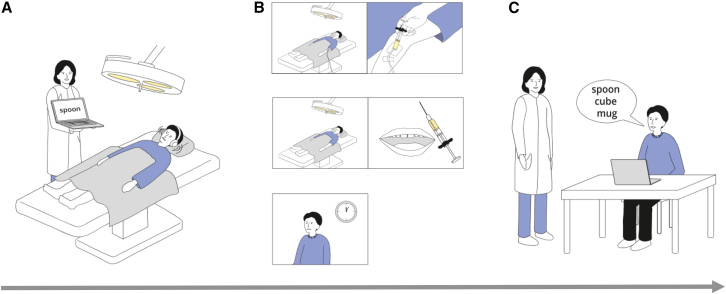
Task and experimental conditions (A) learning phase. While being in a supine position, participants learned a list of words that were related to a single pre-determined schema (“restaurant”) or not. Participants wore headphones that provided a soundscape suggestive of a restaurant. (B) consolidation phase. About 12 min after learning, participants received general anesthesia with propofol for about 60 min (top row), local anesthesia (middle row), or no anesthesia (bottom row). (C) retrieval phase. About 3 h after learning, participants were tested for the recall and recognition of words.

During the learning phase, word lists were presented repeatedly in three learning blocks with an equal number of randomized schema-related and -unrelated words (see Figure 1 for experimental protocol).

All groups showed learning of word lists across the three learning blocks with a continuous increase of recalled words (Χ^2^(2) ≥ 36.4, *p* < 0.001, Friedman ANOVA, Table 1). The increase from block 1 to block 3 was significant in all groups (Z ≤ - 1.72, *p* < 0.001, Wilcoxon signed ranks test). However, the percentage of learned words differed between groups within learning blocks and was significantly different in blocks 1 and 3 (block 1: Χ^2^(2) = 9.06, *p* = 0.011; block 2: Χ^2^(2) = 5.06, *p* = 0.08; block 3: Χ^2^(2) = 8.47, *p* = 0.014, Kruskal-Wallis ANOVA). Post-hoc testing showed that these differences were mainly due to differences between the propofol group and the no anesthesia group, with inferior performance of the propofol group (block 1: Z = −2.95, *p* = 0.003; block 3: Z = −2.65, *p* = 0.008; Mann-Whitney test). Performance between the propofol group and the local anesthesia group did not differ significantly (block 1: Z = −1.29, *p* = 0.2; block 3: Z = −2.95 *p* = 0.28; Mann-Whitney test). We suppose that presurgical arousal or other surgery-related contextual factors may have affected learning in patients, as it has been shown previously that learning under stress can impair subsequent memory of stressor-unrelated information.^34^^,^^35^

**Table 1:** Performance of subject groups during learning blocks in percent correct

	Block 1	Block 2	Block 3	p
Propofol	22.5 (20.0–32.5)	42.5 (27.5–47.5)	42.5 (30.0–62.5)	<0.001
No anesthesia	32.5 (25.0–42.5)	50.0 (32.5–72.5)	62.5 (55.0–75.0)	<0.001
Local anesthesia	27.5 (22.5–35.0)	40.0 (35.0–52.5)	52.5 (42.5–62.5)	<0.001

Values are medians and interquartile ranges. *p*-values refer to Friedman-ANOVA.

**Figure 2:**
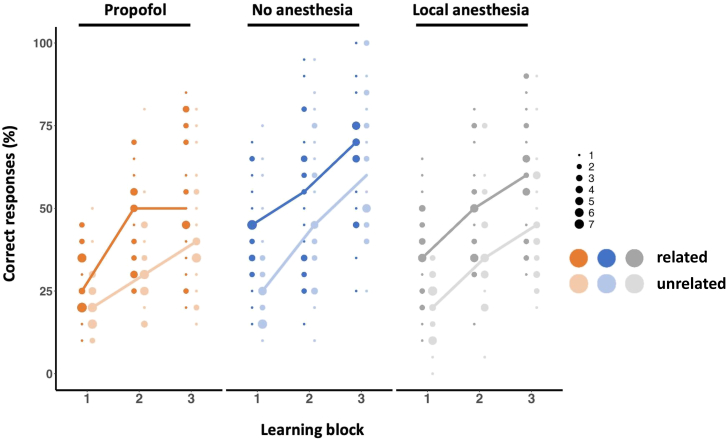
Performance of subject groups during learning blocks in percent correct responses Performance separately for schema-related words (dark dots) and schema-unrelated words (light dots). Dot size shows the number of identical values. Lines connect median values. Note increase in performance from block 1 to block 3 in all groups. Note superior performance for schema-related words compared to schema-unrelated words in all groups.

However, when analyzed separately, schema-related words were significantly better learned than schema-unrelated words in all groups (performance averaged across blocks; propofol group: Z = - 3.55, *p* < 0.001; no anesthesia group: Z = - 2.26, *p* = 0.024; local anesthesia group: Z = - 3.72, *p* < 0.001; Wilcoxon signed ranks test; Figure 2). Since schema-related and -unrelated words were matched in terms of word length, emotional valence, arousal, imageability, and frequency of occurrence in the German language, these differences suggest that the contextual factors of our paradigm reliably activated knowledge from previous experience that supported learning of new schema-related information in all participant groups.

### Recall

All participant groups recalled on average at least 30% of the learned words across the 3-h delay between the end of learning (i.e., block 3) and recall testing, regardless of schema-relatedness (Table 2). The number of false positive words was low in both conditions in all groups (median: 0, IQR: 0–1 in all groups and conditions; schema-related words: Χ^2^(2) = 1.92, *p* = 0.38 difference between groups; schema-unrelated words: Χ^2^(2) = 0.74, *p* = 0.69 difference between groups; Kruskal-Wallis ANOVA).

**Table 2:** Performance of subject groups in the last learning block (block 3) and at delayed recall about 3 h later in percent correct

		Block 3	Recall	p
Propofol	related	50.0 (40.0–75.0)	50.0 (40.0–75.0)	0.821
	unrelated	40.0 (35.0–55.0)	30.0 (15.0–40.0)	0.001
No anesthesia	related	70.0 (50.0–75.0)	60.0 (40.0–65.0)	0.006
	unrelated	60.0 (50.0–80.0)	50.0 (35.0–75.0)	0.003
Local anesthesia	related	60.0 (45.0–75.0)	45.0 (35.0–70.0)	0.001
	unrelated	45.0 (30.0–60.0)	35.0 (25.0–50.0)	0.009

Values are medians and interquartile ranges. *p*-values refer to Wilcoxon signed-ranks test.

However, significant differences were found between performance on block 3 and at recall testing for schema-unrelated words in all groups (Z ≤ −2.6, *p* ≤ 0.009, Wilcoxon signed ranks test). Performance at recall was inferior to block 3 in all groups. Similarly, performance at recall testing was inferior to performance in block 3 for schema-related words in the no anesthesia group and the local anesthesia group (Z ≤ −2.74, *p* ≤ 0.006, Wilcoxon signed ranks test), but not in the propofol group (Z = −0.23, *p* = 0.821, Wilcoxon signed ranks test).

**Figure 3:**
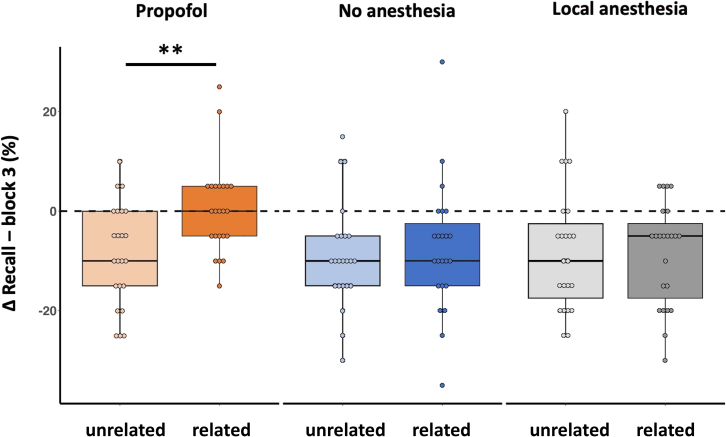
Differences in recall of stimulus words from learning block 3 to recall 3 h after learning, separately for schema-related and -unrelated words for each group Boxplots with medians and inter-quartile ranges. Negative values denote forgetting. Dashed line indicates unchanged performance. Note the similar forgetting of schema-related and schema-unrelated words in the no anesthesia and local anesthesia groups. Note forgetting of schema-unrelated words and preserved memory of schema-related words in the propofol group. ∗∗*p* = 0.001, Wilcoxon signed ranks test.

We then analyzed possible differences in forgetting of schema-related and -unrelated words across the 3-h delay. We subtracted block 3 performance values from recall values, thus yielding negative Δ values for forgetting (Figure 3). In the no anesthesia group and the local anesthesia group, schema-related and -unrelated words were equally forgotten with no significant difference between Δ values (Figure 3, no anesthesia group: Z = −0.53, *p* = 0.6; local anesthesia group: Z = 0.19, *p* = 0.85, Wilcoxon signed ranks test). By contrast, in the propofol group, a significant difference in forgetting between the two stimulus categories was found (Z = −3.27, *p* = 0.001, Wilcoxon signed ranks test) with the forgetting of schema-unrelated words and almost unchanged performance for schema-related words (Figure 3).

Furthermore, while Δ values for schema-unrelated words did not differ significantly between groups, Δ values for schema-related words differed significantly (unrelated: Χ^2^(2) = 0.70, *p* = 0.966; related: Χ^2^(2) = 9.47, *p* = 0.009, Kruskal-Wallis ANOVA). Post-hoc testing showed that Δ values of schema-related words did not differ between the no anesthesia and the local anesthesia group (Z = −0.12, *p* = 0.902; Mann-Whitney test), but differed significantly between the propofol group and the other two groups (propofol – no anesthesia: Z = −2.72, *p* = 0.007; propofol – local anesthesia: Z = −2.60, *p* = 0.009; Mann-Whitney test). In addition to higher Δ values for schema-related words, we found that in the propofol group, the overlap of words during free recall with words recalled in block 3 was significantly higher for schema-related than for -unrelated words (related, 75%; unrelated, 57%; Z = −2.83, *p* = 0.005, Wilcoxon signed ranks test).

Lastly, as can be seen in Figure 3, the effect of propofol on the memory of schema-related words varied across individuals. We therefore separated participants who showed a gain in performance from those who did not and split the propofol group into participants with positive Δ values (i.e., with a gain from block 3 to recall, *n* = 9) and participants with Δ values of zero or below (i.e., with no gain from block 3 to recall, *n* = 14). We found no significant differences between both groups for recall of schema-unrelated words (Z = −1.58, *p* = 0.12; Mann-Whitney test), the recognition of schema-related words (Z = −1.3, *p* = 0.22; Mann-Whitney test), and recognition of schema-unrelated words (Z = −1.14, *p* = 0.28; Mann-Whitney test). This analysis further supports that propofol specifically modulated the consolidation of schema-related items.

### Recognition

Like in a previous study, we found no effect of propofol on the recognition memory of word lists.^27^ Recognition performance and confidence ratings were almost at ceiling in all subject groups, both for schema-related and -unrelated words (Table 3). The observed recall-recognition dissociation might therefore mainly reflect a memory strength confound, i.e., strong memories have been compared to weak memories, rather than a differential impairment of two distinct memory systems.^36^ It is therefore possible that increasing task difficulty would have revealed recognition memory deficits, too.

**Table 3:** Performance of subject groups at recognition testing

		Related	Unrelated	p
Propofol	Recognition (%)	95.0 (90.0–100.0)	95.0 (85.0–100.0)	0.14
	False positives (n)	3.0 (0.0–7.0)	2.0 (0.0–3.0)	0.01
	Confidence (Likert)	3.93 (3.84–4.0)	3.94 (3.84–4.0)	0.614
No anesthesia	Recognition (%)	100.0 (85.0–100)	95.0 (80.0–100)	0.098
	False positives (n)	2.0 (0.0–7.0)	0.0 (0.0–3.0)	0.016
	Confidence (Likert)	3.95 (3.82–4.0)	4.0 (3.78–4.0)	0.753
Local anesthesia	Recognition (%)	95.0 (85.0–100.0)	95.0 (85.0–100.0)	0.273
	False positives (n)	3.0 (2.0–4.0)	1.0 (0.0–2.0)	<0.001
	Confidence (Likert)	3.85 (3.69–3.95)	3.85 (3.72–3.9)	0.398

Values are medians and interquartile ranges. *p*-values refer to Wilcoxon signed ranks test.

In addition, no significant differences were found between the two stimulus categories in all participant groups (recognition: Z ≥ −1.65, *p* ≥ 0.098; familiarity: Z ≥ −0.84, *p* ≥ 0.398, Wilcoxon signed ranks test). Furthermore, although the absolute number of false positive ratings was low, we found significantly more false positive ratings for schema-related words in all subject groups (Table 3; Z ≤ −2.41, *p* ≤ 0.016, Wilcoxon signed ranks test), thus suggesting that schema-dependent predictive processing was still implicitly present in the testing period of our paradigm.^37^^,^^38^

## Discussion

The present study investigated the effects of the anesthetic propofol on the memory consolidation of to-be-remembered words that differ in their relatedness to a predefined schema. For post-anesthesia recall, we found a significant difference between schema-related and -unrelated words. This was mainly due to a benefit for schema-related words uniquely in the propofol group, thus suggesting that propofol facilitated the consolidation of newly encoded schema-related words but not of -unrelated words. Our findings suggest that schema-relatedness modulates memory networks during early memory consolidation. They are further consistent with the hypothesis that schema-related and -unrelated information may take distinct routes for consolidation shortly after learning.

Although not explicitly instructed, the results of the learning trials show that the contextual factors of our paradigm (i.e., the soundscape and the large proportion of stimulus words consistent with a single schema) reliably activated a mental schema that the supported learning of new information. Consistent with the hypothesis that schemas provide a framework that facilitates encoding,^7^^,^^8^^,^^39^ a significant benefit for schema-related words across learning blocks was observed in all three groups. It is unclear whether this finding mainly reflects a quantitative difference in the processing of schema-related and -unrelated information or whether it also reflects distinct trajectories of consolidation recruiting distinct neural substrates. At least in rats, pre-existing schemas have been shown to significantly accelerate the integration of new memoranda into the neocortex with only a transient role of the hippocampus for schema-related information.^6^^,^^40^ In humans, fMRI studies have shown that the availability of a schema significantly modulates connectivity between the hippocampus and ventromedial prefrontal cortex at encoding and during the post-encoding period.^11^^,^^12^^,^^13^^,^^14^ There is, however, no clear behavioral evidence from humans, whether and how schema-relatedness alters the trajectories of consolidation shortly after learning. In particular, it is open whether schema-related information also undergoes accelerated integration into neocortical memory networks.^8^ If so, behavioral consequences of the suppression of neural activity during the consolidation period should be different for schema-related and -unrelated information. The pharmacological manipulation used here used a potent anesthetic that targets GABA-A receptors, i.e., inhibitory receptors that are widely distributed in the brain, including the neocortex and hippocampus.^21^^,^^22^^,^^23^ Accordingly, propofol administration has been shown to inhibit key processes of memory formation in animal experiments.^41^^,^^42^^,^^43^ Likewise, in humans, propofol inhibits hippocampal activation in memory tasks^26^ and impairs the early memory consolidation of verbal and visuospatial stimuli.^27^^,^^28^ The observed differences in free recall of schema-related and -unrelated words after propofol administration thus are consistent with the hypothesis that schema-dependent differences in memory consolidation are not a mere consequence of the facilitated encoding of schema-related information. Rather, they may result from a continuous process that significantly extends into the post-encoding period and that is accompanied by a differential role of memory networks for the consolidation of schema-related and -unrelated information.

The unimpaired retention of schema-unrelated words in the propofol group seems to contradict earlier results from a previous study, where the application of propofol impaired free recall of word lists learned shortly before injection.^27^ However, although both studies used word lists as stimulus material, the mode of the presentation of the lists was significantly different and may have affected how stimuli were encoded and consolidated. In our first study, stimuli consisted of a simple list of words from various semantic categories that was repeatedly presented in a fixed order during learning.^27^ In the present study, lists consisted of words, half of which were related to a pre-defined schema, which were presented pseudo-randomly in unpredictable order during learning. Since the experiments of Donald Hebb, it is known that serial learning benefits significantly from the repeated presentation of stimulus material in a fixed order.^44^ This effect has been observed across a wide range of verbal and visual stimuli, and various associative mechanisms have been proposed to account for it.^45^ In principle, individual items may be associated with a distinct position in the list or with neighboring items. Serial recall experiments suggest that it is more likely that items of a list may be grouped into several chunks of information or into a unified representation of the entire list.^46^^,^^47^ These associative mechanisms are not necessarily mutually exclusive and may have contributed to performance in our first study^27^ but are unlikely in the present study. Experiments with transcranial brain stimulation following visual paired associate learning and with propofol injections following navigational learning suggest that the formation of associations between memory items critically depends on neural activity within a time window of about 60 Minutes after learning.^15^^,^^28^ Therefore, one explanation for the seemingly divergent findings from our first study and the present investigation is that propofol may not have interfered with memory of single items in the first study but rather with associative mechanisms that support memory consolidation in conditions where the stimulus material is learned repeatedly in a fixed order. In support of this hypothesis, studies have shown that the consolidation of the spontaneous associative binding of words in repeated word list learning is impaired in patients with lesions of the hippocampus.^48^ Accordingly, recall performance levels following propofol anesthesia in our first study^27^ are similar to recall before and after propofol anesthesia in the present study.

So why then did we observe better memory for schema-related words rather than impaired memory for schema-unrelated words after the administration of an inhibitory drug? Human studies have shown that various drugs, including alcohol and benzodiazepines, can lead to better verbal and visual memory performance when administered shortly after learning.^49^^,^^50^^,^^51^^,^^52^ One common denominator behind these effects is suggested to be a GABA-A-mediated mechanism, whereby these drugs inhibit the induction of long-term potentiation (LTP) in the hippocampus.^53^^,^^54^ The resulting inhibition of hippocampal encoding would prevent retroactive interference that would otherwise have weakened memories established prior to drug administration.^55^ In line with this hypothesis, a recent study reported effects of intrahippocampal injections of the GABA-A-agonist muscimol on the performance of rats in a novel-object recognition memory task.^56^ About 30 min after encoding, rats received muscimol injections either in the awake state or during sleep. At testing, opposed effects were observed with decreased memory for objects when muscimol was applied during sleep and increased memory when it was applied during the awake state. The authors hypothesized a competitive interaction between a hippocampally mediated memory system and memory systems in extrahippocampal brain regions. In the awake state, consolidation processes in these latter regions might be disturbed by interfering hippocampal activity.^56^ These and the aforementioned findings are consistent with an “opportunistic” hypothesis of memory consolidation, which posits that both synaptic and systems consolidation of newly encoded information are facilitated by subsequent periods of reduced interference – be it by slow-wave sleep or by the application of drugs.^55^

When our results are discussed within the framework of opportunistic memory consolidation, propofol-induced decreased forgetting of schema-related words would mean that – despite presumed the facilitation of neocortical integration – at least some memory network nodes are still relevant for the consolidation of schema-related words in the post-encoding period. This role seems to be inhibitory, at least at this timepoint and in our paradigm. It is tempting to speculate that hippocampal deactivation may have been responsible for facilitated the extrahippocampal integration of schema-related words, while schema-unrelated words did not benefit. Our findings would thus fit the hypothesis that hippocampus-dependent and extrahippocampal memory networks can compete in the post-encoding period.^56^^,^^57^ They would also fit recent findings in rats that the hippocampus exerts an inhibitory influence on neocortical schema learning.^58^ In this study, the deactivation of hippocampal output to “schema cells” in orbitofrontal cortex facilitated the application of an established schema to a new problem in a spatial schema-learning task. Interestingly, this was not behaviorally evident when an already established schema was applied to a familiar situation. How this relates to changes in connectivity between hippocampus and neocortex in human fMRI studies is not clear, as opposing connectivity patterns between hippocampus and ventromedial prefrontal cortex have been reported to be associated with memory of schema-related information in the post-encoding period.^11^^,^^12^^,^^13^^,^^14^^,^^59^

In conclusion, our results show that schema-relatedness affects the early consolidation of verbal information. The pattern of results is consistent with a differential role of memory networks for schema-related and –unrelated information shortly after learning. Our data further suggest that despite accelerated encoding, the integration of schema-related words into presumably neocortical networks seems to significantly extend into the post-encoding period, with an inhibitory role of presumably hippocampus-dependent memory networks. These results thus add support to the concept of distinct and competing memory systems that flexibly interact according to the availability of previous knowledge. However, since GABA-A receptors are widely distributed in the brain, the neural basis of this hypothesis must remain speculative. Once non-invasive hippocampal stimulation techniques are more widely available, their combination with schema-learning tasks and fMRI may ultimately allow for human experiments that directly corroborate this hypothesis.

### Limitations of the study

It should be conceded that the medical setting in which the testing of the patient groups was conducted is an important limitation of our study, as it is likely that both patient groups experienced stress pre-surgically that was not present in the no anesthesia group. Whereas the latter underwent no medical procedures, were tested in a doctor’s office room and were free to ambulate outside the office before testing, the two patient groups were investigated in an outpatient setting for elective minor surgeries of vital regions of the head. Learning in patients was conducted in similar sized rooms/corridors, with visible medical and surgical equipment and the presence of medical staff. In contrast to controls, both patient groups remained in a medical setting until testing. Although we did not measure stress explicitly in our patient groups, previous research with patients undergoing various minor surgical procedures suggests that both global and local anesthesia may cause similar levels of pre-surgical stress and anxiety.^60^^,^^61^^,^^62^ Stress has been shown to impair learning and consolidation of stressor-unrelated material.^34^^,^^35^ Moreover, stress may impair the learning of schema-related information as compared to schema-unrelated information, presumably by altering hippocampal activity and by increasing functional connectivity of the hippocampus with neocortical regions involved in schema-processing.^31^^,^^63^^,^^64^ However, we deem this effect not to be decisive for our main result, as similar effects of schema-relatedness were observed in all three investigated groups. It is nevertheless possible that stress in patients may not only have affected learning but also trajectories and neural substrates of subsequent memory consolidation.^65^ Since our neuro-pharmacological approach only allows for indirect inferences about the brain systems involved, the conclusions of our study should be substantiated by additional imaging experiments.

Furthermore, propofol-induced decreased the forgetting of schema-related words need not necessarily result from the distinct processing of schema-related and -unrelated words. Both stimulus categories may be processed and represented by the same brain systems but may show different levels of semantic interference, with a significant reduction in interference by propofol for schema-related words only. Previous research indeed has shown that semantically related verbal and visual information may interfere in memory, even when presented with unrelated intervening trials.^66^^,^^67^ However, if this factor had been decisive, we would have expected superior learning and memory of semantically unrelated words. We therefore favor the hypothesis of distinct processing modes for both stimulus categories.

Lastly, we would like to point out that the context of being in a hospital likely activates a corresponding schema that may potentially interfere with the activation of an experimentally induced schema that deviates from this context. The more so, as contextual information is encoded and reinstated by hippocampal neurons.^68^^,^^69^ The magnitude of the schema effect observed in our study may thus differ from effects that can be expected in an ideal laboratory setting, where contextual factors can be controlled and are less likely to interfere with experimentally induced schemas.

## Resource availability

### Lead contact

Requests for further information and resources should be directed to and will be fulfilled by the lead contact, Christoph Ploner (christoph.ploner@charite.de).

### Materials availability

Experimental materials consisted of a word list that is provided in the supplemental information of the article.

### Data and code availability

De-identified human data have been deposited in an OSF repository and are publicly available. The DOI is listed in the key resources table. This article does not report original code. Any additional information required to reanalyze the data reported in this article is available from the lead contact upon request.

## Acknowledgments

This study was funded by the 10.13039/501100001659Deutsche Forschungsgemeinschaft (DFG, German Research Foundation), Project number 327654276 - B05, SFB 1315. We thank the study participants and the staff of the Department of Otorhinolaryngology, the Department of Ophthalmology and the Department of Anesthesiology of the Charité – Universitätsmedizin Berlin, Campus Virchow-Klinikum, and Stephan Kinder, Troisdorf, for their kind support of our study. Miriam Seith assisted during the preparation of figures. Special thanks to Lynn Nadel for helpful comments on the article.

## Author contributions

Conceptualization, C.J.P., L.R., and Y.L.S; methodology, C.J.P., L.R., D.I., M.M., C.F., and Y.L.S.; investigation, L.R. and L.L.; writing – original draft, C.J.P. and L.R.; writing – review and editing, C.J.P., L.R., Y.L.S., D.I., and C.F.; funding acquisition, C.J.P. and C.F.; resources, M.M., H.O., and D.J.S.; supervision, C.J.P.

## Declaration of interests

The authors declare no competing interests.

## STAR★Methods

### Key resources table

**Table:** 

REAGENT or RESOURCE	SOURCE	IDENTIFIER
**Deposited data**		

Experimental data	This paper	https://osf.io/gv9zs/

**Software and algorithms**		

IBM SPSS Statistics for Windows, Version 29.0.2.0	Armonk, NY: IBM Corp	https://www.ibm.com
Presentation Version 21.1	Neurobehavioral Systems, Berkeley, CA	https://www.neurobs.com/
R Version 4.1.2	R Core Team	https://www.R-project.org/

### Experimental model and study participant details

A total of 69 participants between 18 and 60 years without any history of neurological or psychiatric disorders, hearing disorders, visual disturbances or substance abuse was included in the study. All participants were native German speakers. Three groups of 23 participants each, matched for sex, age and educational level were tested with a verbal memory task (below Table). One group received general anesthesia with propofol between learning and testing, one group local anesthesia and one group no anesthesia.

**Table:** Demographic and clinical data of the invested patient groups

	Propofol	No anesthesia	Local Anesthesia
n	23	23	23
Female/Male	10/13	13/10	13/10
Age (Years)	37 (21.5–49.5)	39 (25.5–52.5)	41 (29–51)
Years of Education	15 (14–17)	16 (15–18)	15 (14–16.5)
Medical Procedure	Strabismus surgery (*n* = 19) Maxillofacial surgery (*n* = 4)	n.a.	Maxillofacial surgery (*n* = 23)
Propofol Bolus Dose (mg)	200 (200–200)	n.a.	n.a.
Propofol Maintenance Dose (mg/kg/h)	6 (6–6)	n.a.	n.a.
Remifentanil Dose (μg/kg/min)	0.2 (0.2–0.2)	n.a.	n.a.
Time between end of learning and Propofol (min)	12 (9–14.5)	n.a.	n.a.
Duration anesthesia (min)	61 (56–66.5)	n.a.	n.a.
Time between end of anesthesia and testing (min)	124 (120.5–141)	n.a.	n.a.
Time between end of learning and testing (min)	195 (185.5–202)	189 (176–199)	192 (184–202)

Values are medians and interquartile ranges; n.a. not applicable.

The propofol group consisted of participants undergoing general anesthesia with propofol for minor strabismus surgery or minor maxillofacial surgery, such as nasal septum reposition and material removal (table)*.* All patients received the same anesthetic protocol. The no anesthesia group consisted of participants that underwent no surgical or other medical procedures (table). The local anesthesia group consisted of participants undergoing local anesthesia for minor maxillofacial surgery, such as wisdom tooth resection or dent implantation (table). This group was recruited to control for possible pre-surgical arousal effects on memory task performance.^26^

Participants undergoing anesthesia were recruited during preparatory outpatient visits. The no anesthesia group was recruited via the intranet of the Charité - Universitätsmedizin Berlin. Sample size was estimated prior to analysis based on data from previous studies on propofol effects on memory.^27^^,^^28^ All procedures were approved by the ethics committee of the Charité – Universitätsmedizin Berlin (reference EA1_166_21). Every participant gave written consent before participation. While there is no reason to assume sex or gender differences on our outcome measures, our study design precludes assessing whether sex and gender influence the effects of propofol on consolidation of schema-related and -unrelated memory.

### Method details

#### Rationale

To study schema effects on early consolidation of memory, we used a word list paradigm that builds on previous work of schema effects on verbal memory.^10^^,^^30^^,^^31^ In order to relate possible differences between memory of schema-related and –unrelated words unequivocally to their schema-relatedness, we matched stimulus words from these two word categories as closely as possible in terms of number of syllables, word length, emotional valence, arousal, imageability and frequency of occurrence in German language. To this end, we based matching of words across stimulus categories primarily on The Berlin Affective Wordlist-Reloaded (BAWL-R), i.e. a list of German words that provides these variables.^70^^,^^71^ However, since not all stimulus words were included in the BAWL-R, we conducted an additional online survey to assess emotional valence, arousal and imageability of the remaining stimulus words (see below). To match for frequency of occurrence in German language, we used the Korpusbasierte Wortgrundformenliste (DeReWo), a list of German words that provides word frequencies in German language.^72^

In our study, we deliberately restricted the schema-related stimulus material to a single semantic category, as the presurgical setting only allows for a short period of experimentation (15 – 20 minutes). The introduction of multiple semantic categories would have created serious problems for randomization, matching of emotional valence, arousal, imageability and frequency of occurrence in German language of stimulus words and creation of suitable and unequivocal soundscapes. The use of a soundscape was introduced to facilitate the use of a mental schema in an easy and non-stressful way for participants. It has previously been shown that even task-irrelevant sounds improve free recall of stimuli, when sound and stimuli are semantically related.^32^^,^^33^

#### Selection of schema

In order to compare memory of schema-related and –unrelated words, a schema with high imageability and familiarity across participants from various social and cultural backgrounds was needed. We deliberately avoided schema that may relate to individual negative experiences (e.g. school, hospital) or to distinct socio-biographical backgrounds (e.g. farm, forest). To ensure that the schema “restaurant” met criteria for our study, a survey with 22 participants was conducted on nurses, doctors, administrative staff and hospital visitors at the Charité – Universitätsmedizin Berlin (mean age 34 years, SD 11.6). Participants were asked to name the first words that come to their mind when thinking of a restaurant. A total of 183 different words was named by participants (mean: 22 words/participant, SD 4.8). Participants were further asked to rate familiarity and imageability of the term “restaurant” and whether they rate it as positive or negative. All participants stated to be familiar with and having a vivid imagination of a restaurant environment. All but one participant rated the term “restaurant” positively. Due to its high imageability and familiarity as well as its positive emotional valence, we decided to use the schema “restaurant” for our study.

#### Selection of schema-related stimulus words

Out of the total of 183 restaurant-related words named by participants, we selected the 50 most frequently mentioned German nouns with up to two syllables. We then searched the BAWL-R and DeReWo to determine their imageability, emotional valence, arousal, number of letters, word type and frequency of occurrence in German language. Thirty out of 50 words were found. The remaining 20 words were rated in an online survey with 30 randomly selected users of the online platform Prolific (mean age 35 years, SD 10.0).^73^ The survey was similar to the BAWL-R, but focused on emotional valence, arousal and imageability of these words. To assess the comparability between this additional survey and the survey conducted for the BAWL-R, 15 non-schema words that had already been rated in the BAWL-R were added to the new survey. The mean deviation between the 15 words already rated in the BAWL-R compared to their new rating in the online survey was 7,4% (SD 6,7) for emotional valence, 8,4% (SD 4,7) for arousal and 10,4% (SD 6,6) for imageability. The new online survey was thus considered comparable to the BAWL-R.

#### Selection of schema-unrelated stimulus words

We then searched for 50 German nouns with up to two syllables that matched the 50 previously defined restaurant-related words in terms of emotional valence, arousal, imageability, and frequency of occurrence in German language. We therefore searched the BAWL-R and DeReWo databases to find the non-restaurant word with the least deviation for the given variables from each restaurant word. This procedure resulted in a list of word-pairs, each consisting of a restaurant-related word and a matched restaurant-unrelated word.

The 50 word-pairs were then rated regarding their relatedness to the schema “restaurant” on a Likert scale from 1 (no relation to restaurant) to 5 (strong relation to restaurant). The ratings were carried out on Prolific with 30 randomly selected test subjects (15 female, mean age 32 years, SD 8.5). The 40 word-pairs with the most strongly restaurant-related and least restaurant-related words were then used for the final stimulus set (mean ratings restaurant words = 4.75, SD = 0.25; mean ratings non-restaurant words = 1.44, SD = 0.29; *p* < 0.0001).

The final set of words showed no significant differences regarding emotional valence (mean difference on a scale from -3 to 3: 0.18, SD = 0.23; *p* = 0.95), arousal (mean difference on a scale from 1 to 5: 0.13, SD = 0,13 , *p* = 0.89), imageability (mean difference on a scale from 1 to 7: 0.2, SD = 0.16, *p* = 0.7) and frequency of occurrence in German language (mean difference on scale from 0 to 29: 1.23, SD = 1.27, *p* = 0.6). See Table S1 for the complete stimulus set.

#### Behavioral testing

Participants were informed that they should perform a memory task and that they would receive a short additional test three hours later. Participants were not instructed about the purpose of the task and received no information about the semantic categories of the to-be-remembered words. Words were presented visually on a 14-inch notebook computer at a distance of about 60 cm from the subject’s eyes, while participants were in a supine position. Words were composed of white letters against a black background. During the entire learning phase, participants wore noise-cancelling on-ear-headphones and were exposed to a soundscape suggestive of a restaurant. The soundscape was provided to facilitate the use of the schema “restaurant” during encoding of the words. Stimuli were programmed using Presentation software (Neurobehavioral Systems, Berkeley, CA, USA).^74^ All task instructions for behavioral testing were standardized and did not differ between groups.

Before learning, the task was explained with written instructions on the notebook screen. Participants were then allowed to ask questions, until the examiner and participant felt confident about participants’ comprehension of the task. Stimuli were presented in three blocks of 40 trials. Each restaurant and non-restaurant word was presented once in each block. Trial order was varied pseudo-randomly between blocks. During each trial, words were presented for 2000 ms, followed by a fixation cross for 2000 ms. After each block, participants were tested for learning of the words and were asked to freely recall as many words as possible. Responses were recorded for later offline analysis. Participants received no feedback about their performance.

After learning, participants underwent general anesthesia (propofol group), local anesthesia (local anesthesia group) or were free to ambulate in the hospital (no anesthesia group). About three hours after the end of the learning phase, all participants were tested for recall and recognition of the learned words. Testing was conducted with the notebook in a quiet room with participants being in a seated position. No soundscape was presented. Initially, participants were requested to recall the word list and to report all recalled words orally while responses were recorded. Participants received no feedback about their performance. For recognition, participants were presented a list of 80 words. The list consisted of the original list of 40 words, 20 new restaurant-related words and 20 new restaurant-unrelated words in pseudorandom order. Words were presented successively on the notebook screen and participants were requested to decide by keypress whether a word had been part of the initial list or not. Presentation of a word was terminated by the keypress of the participant. In addition, participants were requested to rate the confidence of their decisions on a Likert scale from one (not confident) to four (absolutely confident). Then, a fixation cross was presented for 2000 ms and the next word was presented. Participants received no feedback about their performance.

#### Procedure

In the propofol group, participants performed the learning phase of the task in a preparation room or corridor adjacent to the operating theatre, while being in a supine position. The notebook screen was positioned over the participants’ head to ensure unrestricted and comfortable reading of the stimulus words. After termination of the learning phase, preparation for anesthesia started, participants received a peripheral venous access and had a final check-up talk with the responsible anesthesiologist. Participants were then transferred to the surgical theatre and anesthesia was induced with a bolus of 150 - 250 mg propofol, adjusted to the patient’s weight, followed by a continuous infusion of 6mg/kg/h propofol and 0,2 μg/kg/min remifentanil. The median time between the end of the learning phase and the injection of propofol was 12 minutes (IQR 9 – 14.5, table). During anesthesia, participants underwent surgery and were mechanically ventilated with a laryngeal mask. Median duration of anesthesia was 61 minutes (IQR 56 – 66.5, table). After surgery, participants were transferred to a recovery room where they were observed for about one hour. Post-surgery pain was treated with Paracetamol and Ibuprofen. Finally, participants were transferred to the ward, where they were tested for recognition and recall.

In the no anesthesia group, participants performed the learning, recall and recognition phases of the task in a doctor’s room equipped with an examination couch. Participants were put in a supine position. The notebook screen was positioned over the participants’ head to ensure unrestricted and comfortable reading of the stimulus words. After termination of the learning phase, participants were free to ambulate in the hospital, but were told to come back in 170 minutes to perform the final parts of the task. During this period, participants were not allowed to consume caffeine, drugs or other centrally acting substances.

In the local anesthesia group, participants performed the learning phase of the experiment in the surgical theatre after being prepared for surgery and while being in a supine position. The notebook screen was positioned over the participants’ head to ensure unrestricted and comfortable reading of the stimulus words. After the end of the learning phase, the surgeon and his team entered the room and participants had a final check-up talk. Then, local anesthesia was started with local injections of articain. The median time between the end of the learning phase and local anesthesia injection was 10 minutes (IQR 8 - 12). Post-surgical pain was treated with Ibuprofen. After surgery, participants either waited in a patient lounge or ambulated freely. Participants were told to come back 170 minutes after the end of the learning phase. Similar to the no anesthesia group, the free recall and recognition phases of the task were performed in a doctor’s room equipped with an examination couch.

### Quantification and statistical analysis

Data were analyzed by using IBM SPSS statistics (version 29.0) and visualized by using R (Version 4.1.2).^75^^,^^76^ Memory performance was described as percent correct responses in each subject. For learning and delayed recall, we analyzed the percentage of correctly recalled items from the word list. For delayed recognition, we analyzed the percentage of correctly recognized words and the number of false positive recognitions for each subject. Group averages are given as medians with interquartile ranges. Since Kolmogorov–Smirnov testing showed that the assumption of a normal distribution had to be rejected for most variables, non-parametric statistical testing was used for statistical analysis.^77^^,^^78^ For analysis of within-group differences, we used Friedman-ANOVA and two-tailed Wilcoxon signed ranks tests. For analysis of between-group differences, we used Kruskal–Wallis ANOVA and two-tailed Mann–Whitney tests. Significance was accepted at a *p* < 0.05 level. In addition, since multifactorial data sets are frequently analyzed with parametric ANOVAs, a parametric analysis of the main results was added to the supplement of the manuscript (see supplemental information).

## References

[1] Squire L.R., Genzel L., Wixted J.T., Morris R.G. (2015). Memory Consolidation. Cold Spring Harb. Perspect. Biol..

[2] Dudai Y., Karni A., Born J. (2015). The Consolidation and Transformation of Memory. Neuron.

[3] Barry D.N., Maguire E.A. (2019). Remote Memory and the Hippocampus: A Constructive Critique. Trends Cogn. Sci..

[4] Moscovitch M., Gilboa A. (2022). Has the concept of systems consolidation outlived its usefulness? Identification and evaluation of premises underlying systems consolidation. Fac. Rev..

[5] Bartlett F.C. (1932).

[6] Tse D., Langston R.F., Kakeyama M., Bethus I., Spooner P.A., Wood E.R., Witter M.P., Morris R.G.M. (2007). Schemas and Memory Consolidation. Science.

[7] Van Kesteren M.T.R., Ruiter D.J., Fernández G., Henson R.N. (2012). How schema and novelty augment memory formation. Trends Neurosci..

[8] Hebscher M., Wing E., Ryan J., Gilboa A. (2019). Rapid Cortical Plasticity Supports Long-Term Memory Formation. Trends Cogn. Sci..

[9] Alonso A., Van Der Meij J., Tse D., Genzel L. (2020). Naïve to expert: Considering the role of previous knowledge in memory. Brain Neurosci. Adv..

[10] Packard P.A., Rodríguez-Fornells A., Bunzeck N., Nicolás B., de Diego-Balaguer R., Fuentemilla L. (2017). Semantic Congruence Accelerates the Onset of the Neural Signals of Successful Memory Encoding. J. Neurosci..

[11] Van Kesteren M.T.R., Fernández G., Norris D.G., Hermans E.J. (2010). Persistent schema-dependent hippocampal-neocortical connectivity during memory encoding and postencoding rest in humans. Proc. Natl. Acad. Sci. USA.

[12] Liu Z.-X., Grady C., Moscovitch M. (2017). Effects of Prior-Knowledge on Brain Activation and Connectivity During Associative Memory Encoding. Cereb. Cortex.

[13] Audrain S., McAndrews M.P. (2022). Schemas provide a scaffold for neocortical integration of new memories over time. Nat. Commun..

[14] Guo D., Chen G., Yang J. (2023). Effects of schema on the relationship between post-encoding brain connectivity and subsequent durable memory. Sci. Rep..

[15] Tambini A., D’Esposito M. (2020). Causal Contribution of Awake Post-encoding Processes to Episodic Memory Consolidation. Curr. Biol..

[16] Tambini A., Davachi L. (2013). Persistence of hippocampal multivoxel patterns into postencoding rest is related to memory. Proc. Natl. Acad. Sci. USA.

[17] Titiz A.S., Hill M.R.H., Mankin E.A., M Aghajan Z., Eliashiv D., Tchemodanov N., Maoz U., Stern J., Tran M.E., Schuette P. (2017). Theta-burst microstimulation in the human entorhinal area improves memory specificity. eLife.

[18] Violante I.R., Alania K., Cassarà A.M., Neufeld E., Acerbo E., Carron R., Williamson A., Kurtin D.L., Rhodes E., Hampshire A. (2023). Non-invasive temporal interference electrical stimulation of the human hippocampus. Nat. Neurosci..

[19] Sahinovic M.M., Struys M.M.R.F., Absalom A.R. (2018). Clinical Pharmacokinetics and Pharmacodynamics of Propofol. Clin. Pharmacokinet..

[20] Walsh C.T. (2018). Propofol: Milk of Amnesia. Cell.

[21] Kobayashi M., Oi Y. (2017). Actions of Propofol on Neurons in the Cerebral Cortex. J. Nippon Med. Sch..

[22] Engin E., Benham R.S., Rudolph U. (2018). An Emerging Circuit Pharmacology of GABAA Receptors. Trends Pharmacol. Sci..

[23] Sperk G., Kirchmair E., Bakker J., Sieghart W., Drexel M., Kondova I. (2020). Immunohistochemical distribution of 10 GABAA receptor subunits in the forebrain of the rhesus monkey Macaca mulatta. J. Comp. Neurol..

[24] Eisen A.J., Kozachkov L., Bastos A.M., Donoghue J.A., Mahnke M.K., Brincat S.L., Chandra S., Tauber J., Brown E.N., Fiete I.R., Miller E.K. (2024). Propofol anesthesia destabilizes neural dynamics across cortex. Neuron.

[25] Sun X., Zhang H., Gao C., Zhang G., Xu L., Lv M., Chai W. (2008). Imaging the Effects of Propofol on Human Cerebral Glucose Metabolism Using Positron Emission Tomography. J. Int. Med. Res..

[26] Pryor K.O., Root J.C., Mehta M., Stern E., Pan H., Veselis R.A., Silbersweig D.A. (2015). Effect of propofol on the medial temporal lobe emotional memory system: a functional magnetic resonance imaging study in human subjects. Br. J. Anaesth..

[27] Moon D.U., Esfahani-Bayerl N., Finke C., Salchow D.J., Menk M., Bayerl S., Kempter R., Ploner C.J. (2020). Propofol Modulates Early Memory Consolidation in Humans. eNeuro.

[28] Iggena D., Maier P.M., Häußler S.M., Menk M., Olze H., Larkum M.E., Finke C., Ploner C.J. (2022). Post-encoding modulation of spatial memory consolidation by propofol. Cortex.

[29] Galarza Vallejo A., Kroes M.C.W., Rey E., Acedo M.V., Moratti S., Fernández G., Strange B.A. (2019). Propofol-induced deep sedation reduces emotional episodic memory reconsolidation in humans. Sci. Adv..

[30] Craik F.I.M., Tulving E. (1975). Depth of processing and the retention of words in episodic memory. J. Exp. Psychol. Gen..

[31] Vogel S., Kluen L.M., Fernández G., Schwabe L. (2018). Stress leads to aberrant hippocampal involvement when processing schema-related information. Learn. Mem..

[32] Duarte S.E., Ghetti S., Geng J.J. (2023). Object memory is multisensory: Task-irrelevant sounds improve recollection. Psychon. Bull. Rev..

[33] Packard P.A., Soto-Faraco S. (2025). Crossmodal semantic congruence and rarity improve episodic memory. Mem Cogn.

[34] Quaedflieg C.W.E.M., Schwabe L. (2018). Memory dynamics under stress. Memory.

[35] Schwabe L., Hermans E.J., Joëls M., Roozendaal B. (2022). Mechanisms of memory under stress. Neuron.

[36] Wixted J.T., Squire L.R. (2010). The role of the human hippocampus in familiarity-based and recollection-based recognition memory. Behav. Brain Res..

[37] Hubbard R.J., Rommers J., Jacobs C.L., Federmeier K.D. (2019). Downstream Behavioral and Electrophysiological Consequences of Word Prediction on Recognition Memory. Front. Hum. Neurosci..

[38] Höltje G., Mecklinger A. (2022). Benefits and costs of predictive processing: How sentential constraint and word expectedness affect memory formation. Brain Res..

[39] Sekeres M.J., Schomaker J., Nadel L., Tse D. (2024). To update or to create? The influence of novelty and prior knowledge on memory networks. Phil. Trans. R. Soc. B.

[40] Tse D., Takeuchi T., Kakeyama M., Kajii Y., Okuno H., Tohyama C., Bito H., Morris R.G.M. (2011). Schema-Dependent Gene Activation and Memory Encoding in Neocortex. Science.

[41] Wei H., Xiong W., Yang S., Zhou Q., Liang C., Zeng B.X., Xu L. (2002). Propofol facilitates the development of long-term depression (LTD) and impairs the maintenance of long-term potentiation (LTP) in the CA1 region of the hippocampus of anesthetized rats. Neurosci. Lett..

[42] Nagashima K., Zorumski C.F., Izumi Y. (2005). Propofol Inhibits Long-term Potentiation but Not Long-term Depression in Rat Hippocampal Slices. Anesthesiology.

[43] Takamatsu I., Sekiguchi M., Wada K., Sato T., Ozaki M. (2005). Propofol-mediated impairment of CA1 long-term potentiation in mouse hippocampal slices. Neurosci. Lett..

[44] Hebb D.O., Delafresnaye B.F. (1961). Brain mechanisms and learning.

[45] Araya C., Oberauer K., Saito S. (2024). Hebb repetition effects in complex and simple span tasks are based on the same learning mechanism. J. Exp. Psychol. Learn. Mem. Cogn..

[46] Burgess N., Hitch G.J. (2006). A revised model of short-term memory and long-term learning of verbal sequences. J. Mem. Lang..

[47] Page M.P.A., Norris D. (2009). A model linking immediate serial recall, the Hebb repetition effect and the learning of phonological word forms. Phil. Trans. R. Soc. B.

[48] Grewe P., Neu D., Aengenendt J., Woermann F.G., Mertens M., Bien C.G., Kissler J. (2020). Rhinal and hippocampal contributions to spontaneous inter-item binding and verbal memory recall: Evidence from temporal lobe epilepsy. Cortex.

[49] Parker E.S., Morihisa J.M., Wyatt R.J., Schwartz B.L., Weingartner H., Stillman R.C. (1981). The alcohol facilitation effect on memory: A dose-response study. Psychopharmacology.

[50] Ghoneim M.M., Hinrichs J.V., Mewaldt S.P. (1984). Dose-response analysis of the behavioral effects of diazepam: I. Learning and memory. Psychopharmacology.

[51] Fillmore M.T., Kelly T.H., Rush C.R., Hays L. (2001). Retrograde facilitation of memory by triazolam: effects on automatic processes. Psychopharmacology.

[52] Carlyle M., Dumay N., Roberts K., McAndrew A., Stevens T., Lawn W., Morgan C.J.A. (2017). Improved memory for information learnt before alcohol use in social drinkers tested in a naturalistic setting. Sci. Rep..

[53] Blitzer R.D., Gil O., Landau E.M. (1990). Long-term potentiation in rat hippocampus is inhibited by low concentrations of ethanol. Brain Res..

[54] Del Cerro S., Jung M., Lynch G. (1992). Benzodiazepines block long-term potentiation in slices of hippocampus and piriform cortex. Neuroscience.

[55] Mednick S.C., Cai D.J., Shuman T., Anagnostaras S., Wixted J.T. (2011). An opportunistic theory of cellular and systems consolidation. Trends Neurosci..

[56] Sawangjit A., Harkotte M., Oyanedel C.N., Niethard N., Born J., Inostroza M. (2022). Two distinct ways to form long-term object recognition memory during sleep and wakefulness. Proc. Natl. Acad. Sci. USA.

[57] Poldrack R.A., Packard M.G. (2003). Competition among multiple memory systems: converging evidence from animal and human brain studies. Neuropsychologia.

[58] Zong W., Zhou J., Gardner M.P.H., Zhang Z., Costa K.M., Schoenbaum G. (2025). Hippocampal output suppresses orbitofrontal cortex schema cell formation. Nat. Neurosci..

[59] Bein O., Reggev N., Maril A. (2014). Prior knowledge influences on hippocampus and medial prefrontal cortex interactions in subsequent memory. Neuropsychologia.

[60] Schäffer J., Mehrmann M., Heymann-Schramm S., Werry H., Piepenbrock S. (1988). [Perioperative anxiety and postoperative pain suppression in intraocular operations using general anesthesia and local anesthesia]. Anaesthesist.

[61] Wright J., MacNeill A.L., Mayich D.J. (2019). A prospective comparison of wide-awake local anesthesia and general anesthesia for forefoot surgery. Foot Ankle Surg..

[62] Abd Hamid M.H., Abdullah S., Ahmad A.A., Narin Singh P.S.G., Soh E.Z.F., Liu C.Y., Sapuan J. (2021). A Randomized Controlled Trial Comparing Wide-Awake Local Anesthesia With No Tourniquet (WALANT) to General Anesthesia in Plating of Distal Radius Fractures With Pain and Anxiety Level Perception. Cureus.

[63] Kluen L.M., Nixon P., Agorastos A., Wiedemann K., Schwabe L. (2017). Impact of Stress and Glucocorticoids on Schema-Based Learning. Neuropsychopharmacol.

[64] Vogel S., Kluen L.M., Fernández G., Schwabe L. (2018). Stress affects the neural ensemble for integrating new information and prior knowledge. Neuroimage.

[65] Trammell J.P., Clore G.L. (2014). Does stress enhance or impair memory consolidation?. Cogn. Emot..

[66] Harvey D.Y., Schnur T.T. (2016). Different Loci of Semantic Interference in Picture Naming vs. Word-Picture Matching Tasks. Front. Psychol..

[67] Wei T., Schnur T.T. (2016). Long-term interference at the semantic level: Evidence from blocked-cyclic picture matching. J. Exp. Psychol. Learn. Mem. Cogn..

[68] Sekeres M.J., Moscovitch M., Grady C.L., Sullens D.G., Winocur G. (2020). Reminders reinstate context-specificity to generalized remote memories in rats: relation to activity in the hippocampus and aCC. Learn. Mem..

[69] Coelho C.A.O., Mocle A.J., Jacob A.D., Ramsaran A.I., Rashid A.J., Köhler S., Josselyn S.A., Frankland P.W. (2024). Dentate gyrus ensembles gate context-dependent neural states and memory retrieval. Sci. Adv..

[70] Kuchinke L., Jacobs A.M., Võ M.L.-H., Conrad M., Grubich C., Herrmann M. (2006). Modulation of prefrontal cortex activation by emotional words in recognition memory. Neuroreport.

[71] Võ M.L.H., Conrad M., Kuchinke L., Urton K., Hofmann M.J., Jacobs A.M. (2009). The Berlin Affective Word List Reloaded (BAWL-R). Behav. Res. Methods.

[72] Institut für deutsche Sprache, Programmbereich Korpuslinguistik, Mannheim, Deutschland (2013). Korpusbasierte Wortgrundformenliste DeReWo, v-ww-bll-320000g-2012-12-31-1.0. http://www.ids-mannheim.de/derewo.

[73] (2020). Prolific. https://www.prolific.com.

[74] Neurobehavioral Systems, Inc. https://www.neurobs.com.

[75] IBM Corp (2023). SPSS. Version 29.0. https://www.ibm.com/de-de/products/spss-statistics.

[76] R Core Team (2021). R: A language and environment for statistical computing. https://www.R-project.org/.

[77] Altman D.G. (1990).

[78] Altman D.G., Bland J.M. (2009). Parametric v non-parametric methods for data analysis. BMJ.

