# Supplementary material for: Propofol differentially modulates the consolidation of schema-related and -unrelated memory
Source: iScience. 2025 Aug 21;28(9):113415. doi: 10.1016/j.isci.2025.113415 (PMC12441730; doi:10.1016/j.isci.2025.113415)
Supplement: Document S1. Tables S1–S5 and supplemental methods [file mmc1.pdf]

iScience, Volume *28*

## Supplemental information

# Propofol differentially modulates the consolidation of schema-related and -unrelated memory

**Lukas V. Risse, Deetje Iggena, Lili Landerer, Mario Menk, Heidi Olze, Daniel J. Salchow, Carsten Finke, Yee Lee Shing, and Christoph J. Ploner**

**Supplementary Methods**

**Supplementary table 1**. List of stimulus words in alphabetical order

| **Schema-related: Restaurant-related words** | **Schema-unrelated: Non-restaurant-related words** |
|---|---|
| BAR | BACH |
| BIER | BAHRE |
| BROT | BETT |
| DURST | BLÜTE |
| ESSEN | BRIEF |
| FLASCHE | BRÜCKE |
| GABEL | BRUNNEN |
| GAST | BÜRSTE |
| GERUCH | DAUMEN |
| GEWÜRZ | DUSCHE |
| GLAS | ENKEL |
| HERD | FICHTE |
| HUNGER | GOLD |
| KAFFEE | GUMMI |
| KELLNER | KASPERL |
| KOCH | KLOSTER |
| KORKEN | KUGEL |
| KÜCHE | LINIE |
| LÖFFEL | MAGNET |
| MENÜ | MANTEL |
| MESSER | ORGEL |
| NUDEL | PAKT |
| OBER | PALAST |
| PFANNE | PFERD |
| PFEFFER | PILLE |
| PIZZA | Pinsel |
| RECHNUNG | RINDE |
| REZEPT | RING |
| SALAT | RUBIN |
| SALZ | RÜSSEL |
| SOSSE | SCHAUKEL |
| STUHL | SCHUH |
| SUPPE | SCHWAN |
| TABLETT | SPIEL |
| TASSE | SPINNER |
| TELLER | VEILCHEN |
| TISCH | WANNE |
| TOPF | WÜRFEL |
| TRINKGELD | ZIEGEL |
| WEIN | ZIRKUS |

**Supplementary Analysis**

Currently, there is no established non-parametric multifactorial ANOVA that could be used for non-parametric data. In addition to the non-parametric approach in the main manuscript, we therefore conducted parametric ANOVAs of the main results from the learning phase and the recall delta data.

**Analysis S1: Learning Phase**

**Supplementary table 2.** Repeated Measures ANOVA with factors learning block (block 1, 2, 3), schema-relatedness (related, unrelated) and group (propofol, local anesthesia, no anesthesia):

| Factor(s) | Sum of squares | df | Mean sum of squares | F | p-value |
|---|---|---|---|---|---|
| learning block | 43534.179 | 2 | 21767.089 | 183.701 | < 0.001 |
| schema-relatedness | 10250.242 | 1 | 10250.242 | 56.307 | < 0.001 |
| group | 12371.135 | 2 | 6185.568 | 5.576 | 0.006 |
| learning block * group | 308.213 | 4 | 77.053 | 0.650 | 0.628 |
| schema-relatedness*group | 1201.570 | 2 | 600.785 | 3.300 | 0.043 |
| learning block * schema-relatedness | 279.831 | 2 | 139.915 | 1.891 | 0.155 |
| learning block * schema-relatedness * group | 1285.749 | 4 | 321.437 | 4.344 | 0.002 |

In line with the non-parametric analysis in the main manuscript, the results of the parametric analysis also revealed significant effects of factors learning block, schema-relatedness and group. There was no significant interaction of learning block with group and no significant interaction of learning block with schema-relatedness. However, there was a significant interaction of factors learning block with group and of factors learning block with group and schema-relatedness. We therefore conducted further post-hoc tests, where we compared the

performance differences between schema-related and schema-unrelated words for each block in each group.

**Supplementary table 3.** Post-hoc paired t-tests, comparison of differences between schema-related and schema-unrelated words for each block in each group.

| Group | block | Difference of means | SD | T | df | p-value |
|---|---|---|---|---|---|---|
| Propofol | 1 | 6.09 | 10.22 | 2.856 | 22 | 0.005 |
| | 2 | 13.04 | 16.36 | 3.824 | 22 | < 0.001 |
| | 3 | 11.52 | 14.42 | 3.833 | 22 | < 0.001 |
| Local anesthesia | 1 | 17.61 | 14.91 | 5.662 | 22 | < 0.001 |
| | 2 | 10.22 | 15.19 | 3.227 | 22 | 0.004 |
| | 3 | 14.13 | 16.14 | 4.198 | 22 | < 0.001 |
| No anesthesia | 1 | 13.04 | 14.4 | 4.332 | 22 | < 0.001 |
| | 2 | 2.17 | 17.1 | 0.609 | 22 | 0.549 |
| | 3 | 1.7 | 13.6 | 0.612 | 22 | 0.547 |

The results of these post-hoc tests show that significant differences between schema-related and -unrelated words were principally found in all three groups. However, while this difference was present for all three blocks in the propofol and local anesthesia groups, differences were not significant for blocks 2 and 3 in the no anesthesia group. These results remain significant after Bonferroni-correction of the p-value (0.05 / 9 = 0.0056). Since performance of no anesthesia participants was better than performance of the other two groups and since performance increased across learning blocks, a ceiling effect in no anesthesia participants may account for the interactions found in the ANOVA.

**Analysis S2: Recall**

**Supplementary table 4.** Univariate ANOVA with comparison of recall delta values for schema-related and -unrelated words between groups:

| Factor | Sum of squares | df | Mean sum of squares | F | p-value |
|---|---|---|---|---|---|
| Delta schema-related words | 1110.870 | 2 | 555.435 | 4.693 | 0.012 |
| Delta schema-unrelated words | 9.420 | 2 | 4.710 | 0.039 | 0.962 |

In line with the non-parametric analysis in the main manuscript, the results of the parametric analysis also revealed significant differences of delta values of schema-related words between groups, but not of schema-unrelated words. We then conducted further tests, where we compared the delta values for schema-related and schema-unrelated words in each group.

**Supplementary table 5.** Paired t-tests, comparison of delta values for schema-related and schema-unrelated words in each group.

| Group | Difference of means | SD | T | df | p-value |
|---|---|---|---|---|---|
| Propofol | 9.130 | 10.297 | 4.253 | 22 | < 0.001 |
| Local anesthesia | -0.435 | 13.810 | -0,151 | 22 | 0.881 |
| No anesthesia | 1.304 | 15.016 | 0.417 | 22 | 0.681 |

Like the non-parametric analyses in the main manuscript, the results of these analyses show that schema-unrelated words were similarly forgotten in all three groups. However, while this was also true for schema-related words in the local anesthesia and no anesthesia groups, schema-related words in the propofol group were significantly less forgotten. The results of the paired t-tests remain significant after Bonferroni-correction of the p-values (0.05 / 3 = 0.0167).
